# Spatiotemporal interaction and attribution analysis of ecosystem health and human activities in the Daxing’anling forest–grassland Ecotone, China

**DOI:** 10.3389/fpls.2026.1727672

**Published:** 2026-02-24

**Authors:** Wanlong Pang, Yuexin Zhang, Xiuzhi Ma, Yanan Ma

**Affiliations:** College of Forestry, Inner Mongolia Agricultural University, Hohhot, China

**Keywords:** coupling coordination degree model, ecosystem health, human activity intensity, SHAP model, vigor-organization-resilience-services (VORS) model

## Abstract

**Introduction:**

The Daxing'anling forest-grassland ecotone is a critical ecological barrier in northern China and plays an essential role in maintaining regional ecological security. In recent decades, intensified regional development has increased human–environment conflicts; however, the spatiotemporal feedback mechanisms between ecosystem health (EHI) and human activity intensity (HAI) remain insufficiently understood.

**Methods:**

This study proposes a multi-scale Assessment–Coupling–Driving framework. Ecosystem health was quantified using the Vitality–Organization–Resilience–Service (VORS) model, while HAI was constructed by integrating multi-source socioeconomic and environmental datasets. Spatial statistical analyses and XGBoost-SHAP machine learning were applied to investigate spatiotemporal interactions and driving mechanisms of the human–environment system from 2000 to 2023.

**Results:**

From 2000 to 2023, EHI increased by 25.1%, yet strong spatial heterogeneity persisted. Higher EHI was observed in northern primary forest areas, whereas ecological degradation hotspots were concentrated in the southern agro-pastoral ecotone, largely driven by urbanization and agricultural expansion. HAI increased by 16.3%, exhibiting a high-in-plains and low-in-mountainous-areas spatial pattern, indicating strong topographic constraints on human activities. A significant negative spatial correlation was detected between HAI and EHI (Moran’s I = −0.62). Coupling coordination degree analysis revealed a 6.9% expansion of ecological protection zones with low coordination levels, identifying the region as a major conflict zone requiring urgent governance. Potential conservation areas accounted for 48.7% of the total area, highlighting their importance in maintaining regional ecological security. XGBoost-SHAP results demonstrated nonlinear threshold effects of key drivers. Potential evapotranspiration (PET) and low temperatures suppressed EHI, whereas plant available water enhanced ecological stability. Temperatures above 0°C promoted EHI and, together with high GDP, intensified HAI, while PET exceeding 850 mm inhibited HAI. Temperature and precipitation erosive forces dominated coupling coordination, and GDP exhibited a weak positive effect that became negative after exceeding a critical threshold.

**Discussion:**

The findings reveal clear spatial trade-offs and nonlinear threshold effects governing the coupled evolution of ecosystem health and human activities in the Daxing'anling ecotone. The proposed framework provides a scientific basis for coordinated optimization of ecological protection and regional development through process analysis, threshold identification, and zoning-based regulation.

## 1 Introduction

Ecosystem health refers to the capacity of an ecosystem to withstand external disturbances while maintaining structural stability, functional integrity, and service sustainability (Li et al., 2021). It reflects the region’s response threshold to natural stressors and human disturbances (Zhang et al., 2023). However, high-intensity and unsustainable human activities are significantly altering land cover patterns, leading to biodiversity loss and ecological degradation (Liu et al., 2014). This undermines the ecosystem’s ability to provide essential services, posing direct threats to regional ecological security and human well-being (Dou et al., 2019). Nonetheless, human activities do not always have a purely suppressive effect on ecosystem health. Well-designed interventions aimed at ecological restoration can enhance ecosystem stability (Liu et al., 2018). Therefore, understanding the spatial relationship between ecosystem health and human activity, as well as identifying the underlying driving factors, is critical for promoting harmonious regional development between humans and nature (Rong et al., 2023).

Since Rapport introduced the theory of ecosystem health (Rapport et al., 1998; Wang J. et al., 2022), EHI assessments have evolved from using single-species indicators to adopting multi-dimensional integrated models. This has led to the development of two primary paradigms: species response models and comprehensive evaluation frameworks (Costanza et al., 1997). Species response models indirectly assess ecosystem status and environmental quality by extracting specific biological indicators (Costanza et al., 1997; Huang et al., 2023; Zhao et al., 2019). However, they often fail to account for social factors and exhibit time lags. In contrast, comprehensive frameworks, such as the Pressure-State-Response model(PSR) (Zogaris et al., 2018), the Driving-Pressure-State-Impact-Response model (DPSIR) (Wang et al., 2019; Zhang and Zhou, 2025), the Vitality-Organization-Resilience model (VOR), remote sensing ecological indices, fuzzy entropy-weight methods, integrate both internal ecosystem attributes and external pressures to provide a comprehensive assessment of regional EHI. The Vigor-Organization-Resilience-Services model (VORS) (Wei et al., 2025), which incorporates service provision dimensions, improves the accuracy of representing ecosystem multifunctionality, making it a core tool for diagnosing regional EHI (Wang et al., 2023). For example, Li et al. applied an improved VORS model to analyze the spatiotemporal variation of the EHI in the Wuliangsuhai Basin from 2000 to 2022 (Qi et al., 2023). Their findings revealed how geographic heterogeneity regulates ecological resilience, demonstrating the model’s broad applicability in diverse habitat health assessments. Developing multi-dimensional EHI assessment frameworks remains a central challenge in ecology and sustainable geography. A key aspect of this challenge involves deciphering their nonlinear coupling mechanisms with HAI. This issue is particularly urgent in the study of ecologically fragile regions. Investigating land cover changes driven by human activities and analyzing their ecological impacts is therefore essential to addressing the human-environment conflict. For instance, Qi et al. coupled the HAI with an ecosystem services (ESs) quantification model to reveal the nonlinear suppression of ecological regulation functions due to urban expansion in Harbin (Wolf et al., 2023). Similarly, Wolf et al. assessed the ecological risks associated with HAI changes in the Jajrud protected area of Iran using landscape pattern indices from 1989 to 2019 (Chen et al., 2022). Their work provided spatially targeted strategies for regional ecological restoration. Existing research has predominantly focused on Single-Dimensional land-use transitions, often neglecting the interaction of Socio-Economic and other multi-factorial influences. This gap has hindered accurate analysis of the feedback mechanisms between HAI and EHI. To address this, this study adopts a multi-functional approach to measure HAI, using the VORS model and an enhanced HAI assessment to evaluate both EHI and HAI. To overcome the challenges posed by nonlinear mechanisms and threshold identification, emerging explainable machine learning technologies offer valuable solutions. The XGBoost-SHAP model integrates high-precision predictions from gradient-boosted trees with the SHAP values’ game-theoretic explanation framework, allowing for precise quantification of multi-factor interactions (Das et al., 2023). The key advantage of this approach lies in its ability to analyze nonlinear relationships, identify critical thresholds, and reveal the spatial heterogeneous contributions of driving factors. For example, Wu et al. successfully demonstrated the nonlinear effects of environmental regulation on ecological resilience in the Yangtze River Delta (Zhao et al., 2023), confirming the model’s applicability to analyzing mechanisms in ecologically fragile regions. However, this technology has not yet been applied to human-environment systems in temperate forest-grassland ecotones, particularly in terms of identifying the feedback thresholds between EHI and HAI (Rapport and Singh, 2006).

The Daxing’anling forest-grassland ecotone, a typical representative of the temperate forest-grassland transition zone in northern China, plays an irreplaceable role in preventing the southward spread of sand and dust, regulating regional water cycles, and maintaining biodiversity (Li et al., 2024). Over the past two decades, high-intensity agricultural activities, mineral exploitation, and urbanization have significantly increased HAI in this region, resulting in a series of ecological consequences such as forest fragmentation, grassland degradation, and soil erosion (Wang and Dong, 2024; Feng et al., 2025). Understanding the relationship between EHI and HAI is crucial for enhancing ecosystem quality and improving the ecological environment (Gao C. et al., 2025). However, the relationship between these factors remains unclear, hindering the coordinated optimization of ecological protection policies and land management strategies (Bowen et al., 2025; Jing et al., 2025). To address this, this study focuses on the Daxing’anling forest-grassland ecotone from 2000 to 2023 and develops a progressive Assessment-Coupling-Driving analysis framework. The VORS model is employed to quantify the EHI and explore its spatiotemporal evolution. Rates of land use change and human activity interference intensities are integrated to create a multi-dimensional HAI (Das et al., 2021). Spatial autocorrelation and quadrant models are used to analyze the spatial correlation between EHI and HAI. Coupling coordination degree models assess the degree of coordination between EHI and HAI. Additionally, the SHAP explainable machine learning model is introduced to overcome the limitations of traditional linear models, enabling multi-dimensional analysis of the complex nonlinear coupling relationship between EHI and HAI in the temperate forest-grassland ecotone. This method identifies the threshold effects of key driving factors (Wu et al., 2021; Yang and Huang, 2021). The findings provide spatial regulatory guidelines for regional ecological protection and sustainable land resource use, offering valuable methodological insights for the collaborative governance of human-Environment systems in similar ecological transition zones (Ma et al., 2024).

## 2 Study area and data

### 2.1 Study area

**Figure 1:**
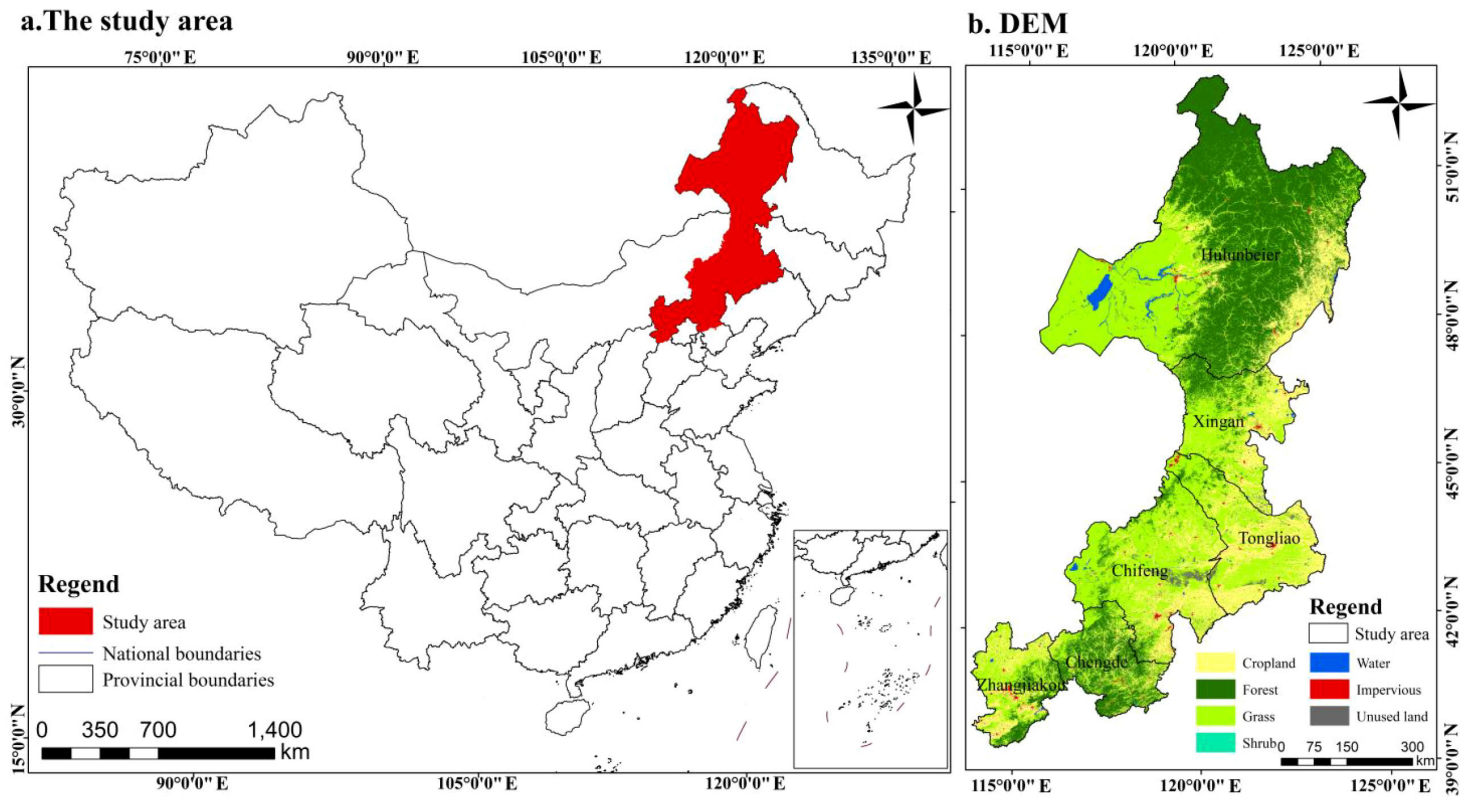
Location of the study area [**(a)** location of forest - grass ecotone in China, **(b)** Land use status].

In this study, we focus on the Daxing’anling forest–grassland ecotone, which is mainly distributed in the Inner Mongolia Autonomous Region., where a clear and continuous forest–grassland transition is observed. Therefore, the term “Daxing’anling forest–grassland ecotone” is used throughout the manuscript for clarity and spatial consistency. The Daxing’anling forest–grassland ecotone (39°30′–53°20′N, 113°50′–126°04′E) covers approximately 520, 000 km^2^, spanning 67 counties and districts, mainly across Hulunbuir and Xing’an League in Inner Mongolia, as well as Zhangjiakou and Chengde in northern Hebei Province (**Figure 1**). The region is located at the transition between the Inner Mongolian grasslands to the west and the Daxing’anling forest region and Songliao Plain agricultural zone to the east, forming a distinct grassland–forest–cropland landscape gradient. The terrain shows a stepwise elevation pattern from west to east and consists primarily of mountains, hills, and plains. The climate is transitional between a temperate semi-humid monsoon climate and a semi-arid continental climate, with an average annual temperature ranging from −6°C to 8°C and mean annual precipitation between 300 and 600 mm, increasing from west to east. Soil and vegetation types display clear zonal differentiation along the west–east gradient. Typical grasslands and sandy Larix forests dominate the western part, while temperate coniferous and broadleaf mixed forests and meadow grasslands prevail in the eastern part. In the transitional zone, multiple ecosystem types intermix, resulting in complex but continuous forest–grassland transition characteristics.

### 2.2 Data source and processing

This study integrates multi-source data to explore the mechanisms underlying ecosystem evolution in the Daxing’anling forest-grassland ecotone. It incorporates various multidimensional indicators, including land use, topography, meteorology, vegetation, soil, human activity, and socio-economic factors. Land-use data were obtained from the China Land Cover Dataset (CLCD) at 30 m resolution, developed by Yang Jie and colleagues at Wuhan University. The latest version covering the period 2000–2023 was downloaded from the official repository and used to derive annual land-use transitions in this study (Gao et al., 2022). The digital elevation model (DEM) was sourced from the Geospatial Data Cloud platform (https://www.gscloud.cn/). Meteorological data were provided by the China Meteorological Science Data Center (https://data.cma.cn/), and soil data were derived from the Chinese subset of the World Soil Database (HWSD). Annual mean vegetation net primary productivity (NPP) and Normalized vegetation index (NDVI) data were sourced from the MOD17A3 and MOD13Q1 satellite products on the Google Earth Engine (GEE) platform. These datasets have a temporal resolution of 8 days and a spatial resolution of 500 m. Potential evapotranspiration (PET) was calculated using the FAO-56 Penman–Monteith equation based on meteorological observations and then spatially interpolated to a 1 km resolution using the ANUSPLIN software, which accounts for topographic effects. HAI was quantified using 1 km nighttime light data from the National Earth System Science Data Center (https://www.geodata.cn/), along with population density and GDP raster data from WorldPop (https://hub.worldpop.org/). Socio-economic statistical data were extracted from the China County Statistical Yearbook and relevant national economic and social development bulletins for the respective districts. The time span of all datasets used in this study is from 2000 to 2023. To ensure spatial consistency, the projection coordinate system was standardized to WGS_1984_UTM_Zone_50N, and all data were resampled to a 1 km resolution. Further details are provided in **Table 1**.

**Table 1:** Main data sources.

Data type	Data description	Data source
Geographic basic data	Data on the scope of administrative districts and counties and rivers	http://www.ngcc.cn/ngcc/
Vegetation data	Vegetation index and productivity data 500m resolution	https://earthengine.google.com
Soil Data	Data sets of soil texture, soil erosion and available water content of plants	http://www.geodata.cn and World Soil Database(HWSD)
Elevation data	Digital elevation and aspect with 30m resolution	http://www.gscloud.cn
Meteorological data	Annual precipitation and precipitation erosion 1km dataset	http://www.data.cma.cn
Land use data	CLCD30m land use data of Wuhan University	https://zenodo.org.cn
Socio-economic statistics	Population density and Gross Domestic Product (GDP) 1km dataset	http://hub.worldpop.org/
	Night light data(NTL)	http://www.geodata.cn
	Grain output of each city from 2000 to 2023	county-level statistical yearbooks and socio-economic development bulletins.

## 3 Materials and methods

### 3.1 Overview of the study workflow

**Figure 2:**
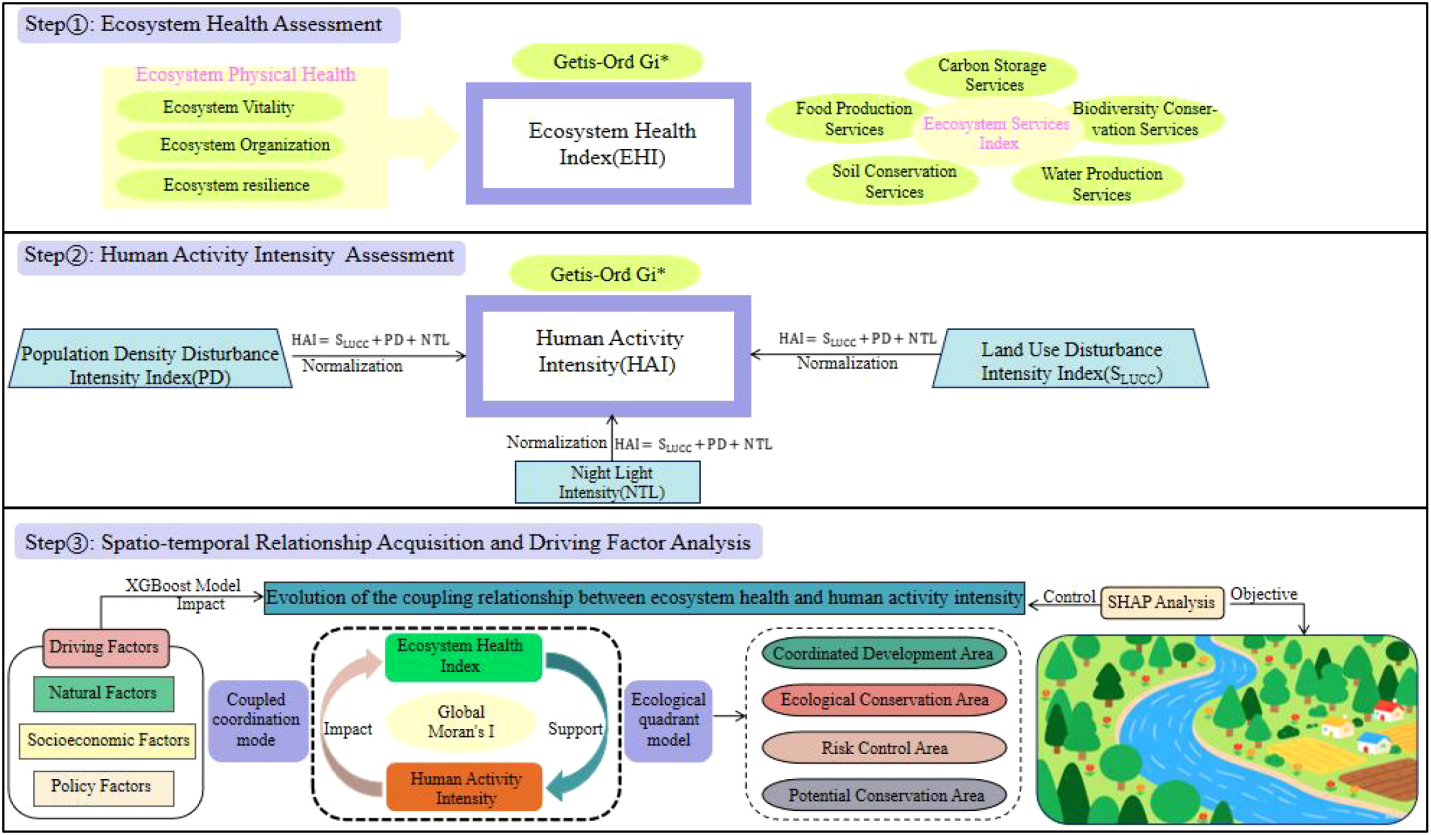
Overview of the study workflow.

This study investigates the interaction between EHI and HAI within the study area. A research framework was developed, consisting of four key steps (**Figure 2**): The EHI was developed by integrating both the physical health of ecosystems and ecosystem services. Subsequently, the HAI was calculated through the normalization and summation of indicators such as population density, land use, and nighttime light disturbance. To analyze the coupling evolution between EHI and HAI, bivariate spatial autocorrelation models (Global Moran’s I), quadrant analysis, and coupling coordination models were applied, enabling the identification of functional zones including coordinated development areas and ecological protection regions. Finally, XGBoost regression and SHAP analysis were employed to uncover the driving mechanisms—natural, socio-economic, and policy factors—behind the coupling relationship, thereby providing valuable insights for regional ecological management.

### 3.2 Ecosystem health assessment methods

EHI was developed using the VORS model, which incorporates vitality, organization, resilience, and ecosystem services. The index provides a comprehensive assessment of the structure–function relationship in the northeastern forest–grassland ecotone, based on two dimensions: ecosystem physical health (EPH) and the ecosystem services index (ESI). Ecosystem physical health (EPH) comprises three components: vitality (EV), organization (EO), and resilience (ER) (Xiao et al., 2019; Wei et al., 2024). Vitality (EV) represents the metabolic capacity or primary productivity of ecosystems and is measured by normalized NPP. Organization (EO) describes ecosystem stability, evaluated through landscape connectivity, heterogeneity, and species richness. Resilience (ER) refers to the ability of ecosystems to resist disturbances and return to their original state and is calculated using area-weighted resistance and recovery coefficients of different land-use types. The ecosystem services index (ESI) was generated using the InVEST model. Five ecosystem service indicators—carbon storage, food production, Environmental quality, soil retention, and water yield—were selected based on regional ecological characteristics. The selection of these ecosystem service indicators and their calculation methods follows a previously established and peer-reviewed framework developed for the Daxing’anling region (Pang et al., 2024), in which the ecosystem service functions were systematically quantified and validated under land-use change. In the present study, the same indicator definitions, model configurations, and parameter settings were adopted to ensure methodological consistency and scientific reliability, rather than redefining ecosystem service functions. Each ecosystem service was quantified using standardized, model-based methods to represent service supply capacity. Indicator weights for ESI were determined using the entropy weight method, an objective weighting approach that assigns weights based on the information variability of each indicator. This method has been widely applied in ecosystem service and ecosystem health assessments and helps reduce subjectivity in parameter assignment. All parameters and weight settings were derived from validated literature or authoritative datasets, ensuring that no arbitrary calibration was introduced in this study. Finally, EHI was derived by integrating EPH and ESI, revealing the synergy between ecosystem physical condition and service provision (Yan Y. et al., 2025; Peng et al., 2018). The detailed calculation procedures, parameter values, and weight settings are presented in **Tables 2** and **3**.

**Table 2:** Methods for quantifying ecosystem health indicators.

Indicator	Formula	Indicator meaning
EHI	$EHI=\sqrt[{4}]{EV\times E0\times ER\times ESI}$	EO, EV and ER are ecosystem organizational force, vitality and resilience, and ESI is ecosystem service index
EV	NPP(x,t)=APAR(x,t)×ϵ(x,t)	APAR(x), represents the photosynthetically active radiation absorbed by pixel x in month t (MJ/m2/month); ϵ(x), represents the actual light energy utilization rate of pixel x in month t (gC/MJ)
EO	EO = 0.3001LC + 0.4366LH + 0.2633IC LC = 0.1499 ×SHDI + 0.1502 ×SHEI LH = 0.1002 ×DIVISION + 0.2173 × IJI + 0.1191×CONTAG IC = 0.2633 × FRAC	LC stands for landscape connectivity; LH is landscape heterogeneity; IC is landscape form; SHDI and SHEI are Shannon diversity index and evenness index; DIVISION, IJI and CONTAG are landscape separation, walking and juxtaposition index and landscape spread degree respectively. FRAC is the fractal dimension; The selected landscape pattern indices were calculated using Fragstats 4.3 and the weights of each index were determined by the combined weighting method
ER	$ER=\sum_{i=1}^{n}A_{i}\times \left(0.4C_{resis\tan t}+0.6C_{resilient}\right)$	A_i_ represents the area of land use type i: $C_{resis\tan t}$and $C_{resilient}$and are the resistance coefficient and resilience coefficient respectively. Through literature combined with the land use and ecological conditions of the Greater Khingan Range, the relevant coefficients are shown in **Table 2**
ESI	$ESI=\sum_{i=1}^{n}P_{i}\times w_{i}$	P, the value of ecosystem service i; m represents the weight of ecosystem service i. The average weight is taken from the literature. The relevant calculation method of ecosystem service P is detailed in the literature^35^

**Table 3:** Ecosystem resilience coefficients.

Land cover types	Farmland	Forest land	Grassland	Waters	Constructions	Desert
Resilience coefficient	0.4	0.9	0.7	0.8	0.2	0.1
Resistance coefficient	0.6	1	0.6	0.8	0.3	0.2

### 3.3 Human activity intensity assessment methods

Research on HAI has evolved from non-spatial statistical approaches to spatially explicit methodologies (Wang et al., 2024). Early studies primarily relied on traditional statistical techniques (Peng et al., 2017), later expanding to include spatially explicit models such as land use change analysis and the Human Footprint Index (HFI). These approaches, however, suffer from limitations in spatial resolution and temporal consistency of indicators (Yan L. et al., 2025). This study addresses these gaps by integrating multi-source data: land use/land cover (LUCC) datasets, nighttime light imagery, and gridded population density. HAI is constructed using a hybrid framework and achieves refined characterization of internal regional differences through spatial weighted analysis (Equation 1):

(1) $$HAI=S_{LUCC}+PD+NTL$$

SLUCC quantifies land-use disturbance intensity via a land type-specific weighting scheme, with assigned coefficients set as follows: construction land = 10, cultivated land = 7, grassland = 3, and other land types = 0. These coefficients represent an ordinal ranking of relative anthropogenic disturbance intensity rather than absolute physical measurements, and reflect the increasing degree of human modification across land-use types. This weighting approach is consistent with existing LUCC-based HAI studies that adopt differentiated coefficients to characterize varying levels of anthropogenic disturbance (Yan L. et al., 2025; Peng et al., 2017; Jing et al., 2025). In addition to SLUCC, the Population Density Disturbance (PD) index is employed to measure the disturbance degree of human settlements, while Nighttime Light (NTL) intensity is used to reflect the intensity of anthropogenic activities. All these indices undergo range normalization prior to spatial weighting calculations.

### 3.4 Spatial analysis methods

This study is based on the ArcGIS platform and employs Global Moran’s I to evaluate the spatial autocorrelation characteristics of the EHI and HAI in the forest-grassland ecotone of Northeast China. The spatial agglomeration patterns of the two indices are identified through cold and hot spot analysis (Getis-Ord Gi*). Finally, the coupling coordination degree model is used to quantify the interaction intensity between EHI and HAI, thereby revealing the mutual promotion or restraint relationships between EHI and HAI. The specific formulas are presented in **Table 4**.

**Table 4:** Methodological details of spatial analysis.

Method	Relevant formula	Parameter definition
Global Moran index	$I=\frac{n\sum_{i=1}^{n}\sum_{j=1}^{n}W_{ij}(x_{i}-\bar{x})(x_{j}-\bar{x})}{\sum_{i=1}^{n}\sum_{j=1}^{n}W_{ij}\sum_{i=1}^{n}(x_{i}-\bar{x}{)}^{2}}$	X_i_ and X_j_ represent the ecosystem health or human activity intensity of study units i and j respectively, $\bar{X}$ is the mean values of the study areas;W_ij_ is the spatial weight matrix;n is a grid cell, I>0, is a positive correlation, is a negative correlation:I=0, is an independent random distribution
Getis-Ord Gi*^40^	$G_{i}^{*}=\frac{\sum_{i=1}^{n}a_{i}Q_{ij}-\bar{a}\sum_{i=1}^{n}Q_{ij}}{\left(\sqrt{\frac{\sum_{i=1}^{n}{a_{i}}^{2}}{n-1}}{\bar{a}}^{2}\right)\sqrt{\frac{n\sum_{i=1}^{n}Q_{ij}^{2}-{\left(\sum_{i=1}^{n}Q_{ij}\right)}^{2}}{n-1}}}$	Gi* is the aggregation index of grid i; a is the attribute value of grid i; Q is the weight matrix; n is the total number of grids; ā represents the mean of the ecosystem health or human activity intensity of all grid cells.
Coupling coordination degree mode	$D=\sqrt{C\times T}$ $C=\frac{2\sqrt{EHI\times HAI}}{EHI+HAI}$ T=αEHI×βHAI	D denotes the coupling coordination degree between the EHI and HAI, calculated based on the coupling degree (C) and a coordination index (T). Here, T is derived from EHI and HAI using weighting coefficients α and β (both set to 0.5, satisfying α + β = 1). The value of D, normalized to the range [0, 1], reflects the intensity of interaction between the two systems: higher values indicate stronger coordination. Following established classification criteria, D is categorized into five levels: severe dissonance (0.00–0.20), mild dissonance (0.20–0.40), near-dissonance (0.40–0.60), mild coordination (0.60–0.80), and high coordination (0.80–1.00).

### 3.5 Ecological quadrant model partitions

The Ecological Quadrant Model, adapted from the Boston Matrix in management science (Pan et al., 2019; Ye et al., 2023), has been widely applied in recent years to ecological risk assessment, landscape security analysis, and evaluations of ecosystem service trade-offs. By dividing space into quadrants defined by two indicators, the model identifies synergistic and conflicting states within ecological–social systems (Levrel et al., 2009; Chen et al., 2013). This approach supports the formulation of targeted governance strategies. In this study, the model was applied with the EHI and HAI as dual indicators. The framework was used to analyze spatiotemporal patterns of human–environment interactions in the northeastern forest–grassland ecotone, an area of ecological fragility (Xia et al., 2025). EHI and HAI were first standardized with Z-scores to remove dimensional differences. The standardized mean values were then applied as thresholds to separate each variable into high and low intervals, producing a four-quadrant coupling map. Quadrant I (coordinated development zone) denotes areas where human activities and ecological functions are temporarily aligned but may exert potential pressure. Quadrant II (ecological management zone) identifies degradation hotspots where human activity substantially exceeds the ecological carrying capacity. Quadrant III (risk prevention zone) describes areas of low EHI driven by the combined effects of natural stress and ecological vulnerability. Quadrant IV (potential conservation zone) denotes areas with favorable natural conditions and high conservation value (Gao RZ. et al., 2025; Zhou and Li, 2025).

### 3.6 Analysis of model-driven factors

(1)XGBoost machine learning algorithm.

In this study, the XGBoost (Extreme Gradient Boosting) machine learning algorithm was adopted to analyze the coupling mechanism between EHI and HAI in the forest-grassland ecotone of Northeast China (Yu and Li, 2024). XGBoost is based on a gradient boosting framework, which gradually optimizes prediction accuracy through the iterative integration of decision tree models via additive learning. Its core advantages include: A second-order Taylor expansion loss function to enhance the convergence efficiency of gradient descent; A regularization strategy that effectively suppresses overfitting; Parallelized feature partitioning and adaptive handling of missing values, enabling adaptation to multi-source heterogeneous ecological data. Algorithm parameters are tuned using grid search and cross-validation, and the contribution of driving factors is quantified in combination with the SHAP (Shapley Additive Explanations) model to reveal the nonlinear action mechanisms of key variables (Equation 2).

(2) $$L^{\left(t\right)}=\sum_{i=1}^{n}\left[g_{i}f_{t}\left(x_{i}\right)+\frac{1}{2}h_{i}f_{t}^{2}\left(x_{i}\right)\right]+\Omega \left(f_{k}\right)$$

L(t) represents the expression in the linear space, i represents the i-th sample, k represents the KTH tree, represents the predicted value of the i-th sample xi, and gi represents the first derivative of the data point on the loss function. hi represents the second derivative of the data point on the loss function.

(2) Explanation of SHAP model factors.

In this study, the SHAP (Shapley Additive Explanations) interpretability model is introduced. Based on the Shapley value principle from cooperative game theory (Equation 3), it quantifies the nonlinear contributions of multi-source driving factors in XGBoost prediction results (Hao et al., 2022; Wang Z. et al., 2022).

(3) $$\varphi_{i}=\sum_{S\subseteq N\backslash \{i\}}\frac{\left|S\right|!\left(n-\left|S\right|-1\right)!}{n!}\left(v\left(S\cup \{i\}\right)-v\left(S\right)\right)$$

In the formula, N={1, 2,… n) represents the set of all features, S represents a subset of N that does not contain feature i, S represents the number of elements in subset S, and v(S) represents a function representing the model predicted value corresponding to the feature combination in subset S.

(3) Accuracy evaluation.

The model accuracy is evaluated through the determination coefficient (R), root mean square error (RMSE), and mean absolute error (MAE) (Equation 4), and the calculation formula is as follows:

(4) $$\begin{array}{l} R^{2}=1-\frac{\sum_{i=1}^{n}{\left(y_{i}-{\hat{y}}_{i}\right)}^{2}}{\sum_{i=1}^{n}{\left(y_{i}-\hat{y}\right)}^{2}}\\ RMSE=\sqrt{\frac{1}{n}\sum_{i=1}^{n}{\left(y_{i}-{\hat{y}}_{i}\right)}^{2}}\\ MAE=\frac{1}{n}\sum_{i=1}^{n}\left|y_{i}-{\hat{y}}_{i}\right| \end{array}$$

n represents the sample size, yi represents the true value, i represents the predicted value, and i represents the average value of the true value yi. It measures the degree of fitting of the model to the data, and its value range is [0, 1]. The closer it is to 1, the better the fitting effect of the model.

## 4 Results and analysis

### 4.1 Spatial and temporal changes in ecosystem health

**Figure 3:**
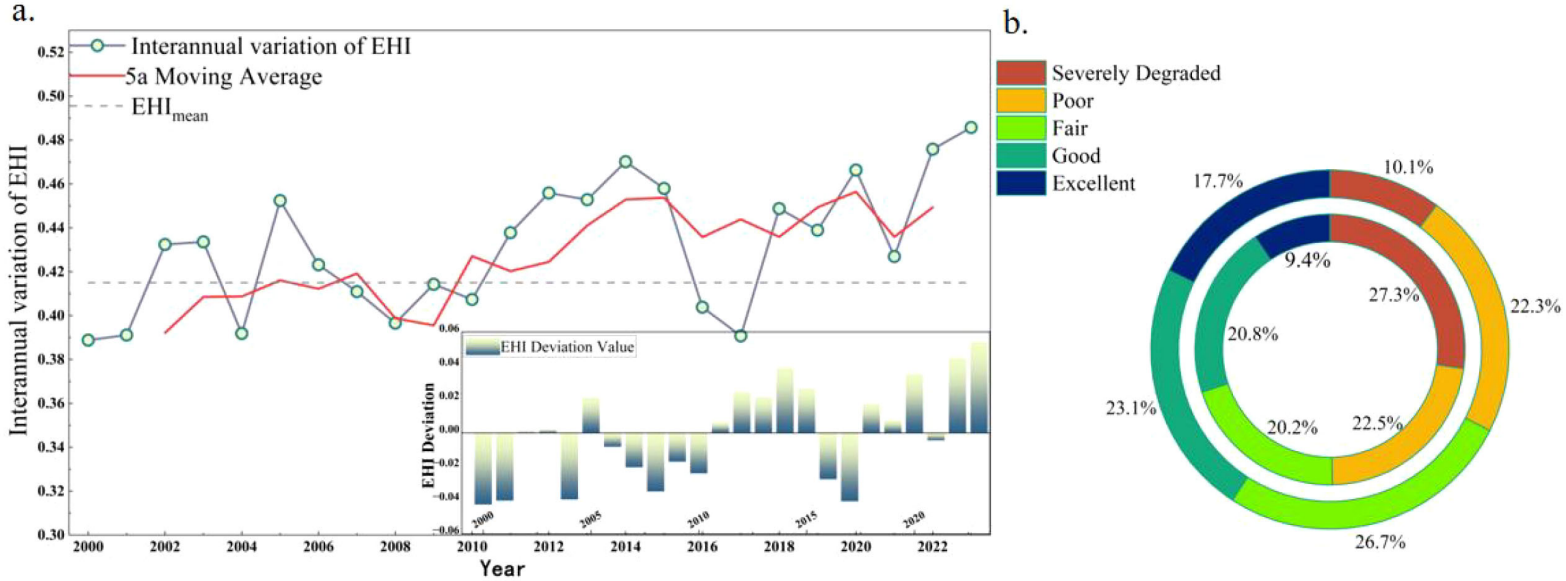
Temporal variation of EHI in the study area from 2000 to 2023 **(a)** is the interannual variation trend diagram of EHI index; **(b)** is the circular diagram of the area ratio of EHI grades in 2000 and 2020.

The EHI gradually improved in the region from 2000 to 2023 (**Figure 3**), the overall EHI rose from 0.388 in 2000 to 0.485 in 2023. Between 2000 and 2008, EHI fluctuated with minimal overall improvement, whereas it showed a clearer upward trend from 2008 to 2016. After 2016, fluctuations continued, but EHI stabilized at a relatively high level. The proportion of areas with poor or very poor EHI declined from 49.8% to 32.4%. These results suggest that EHI in the Daxing’anling forest–grassland ecotone has improved, indicating significant progress in ecological conditions.

**Figure 4:**
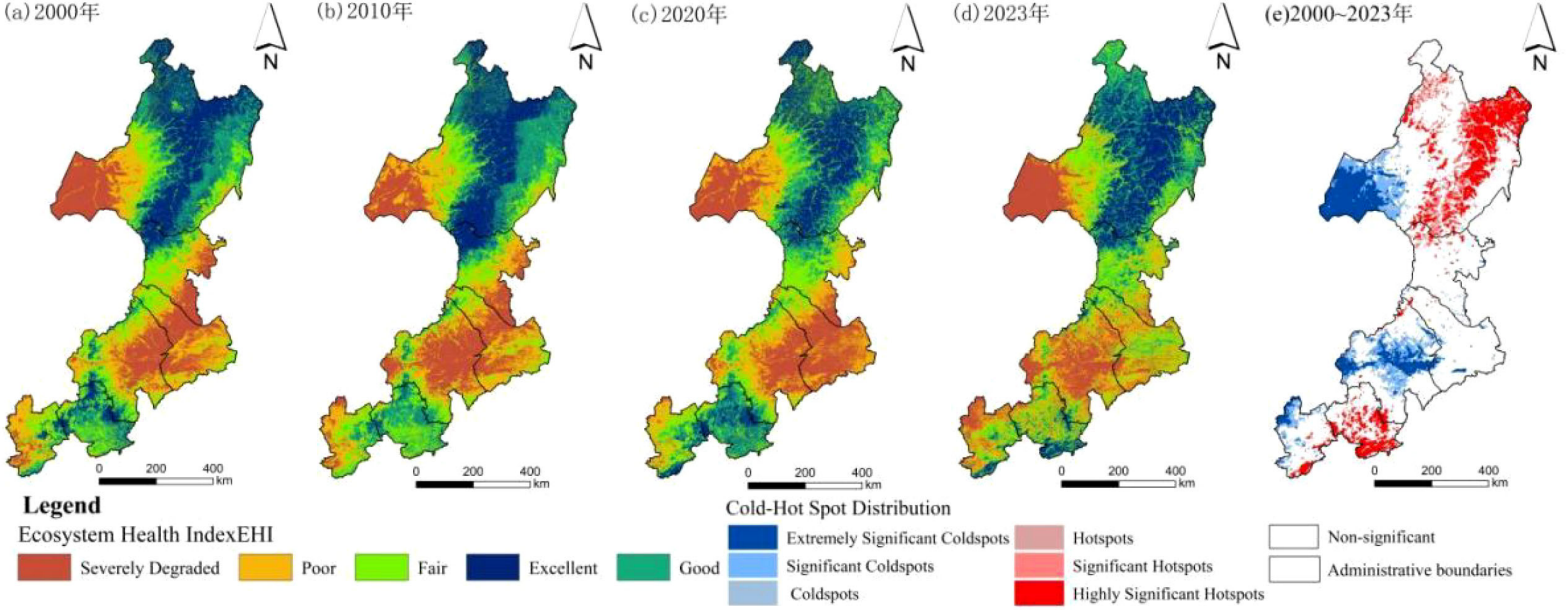
Spatial variation of EHI in the study area from 2000 to 2023.

The EHI in the northeastern forest–grassland ecotone showed clear spatial heterogeneity (**Figure 4**). Areas with high or relatively high EHI were primarily located in the northern Daxing’anling region, where extensive forests and grasslands contribute to stronger EHI. In contrast, low and very low EHI values were concentrated in the central and northwestern Daxing’anling, as well as in densely populated urban centers such as Tongliao, Chifeng, and western Hulunbuir. These regions contained a large proportion of degraded ecosystems. The spatial significance of EHI variation was assessed using the Getis-Ord Gi* statistic. Significant decreases were concentrated in transitional zones between forests and grasslands, as well as in southern urban clusters (cold spots, significance ≥95%). Significant increases were mainly observed in the northern and southeastern parts of the study area, especially in the Daxing’anling primary forest and the Saihanba protected area (hot spots, significance ≥95%).

### 4.2 Spatiotemporal variation of human activity intensity

**Figure 5:**
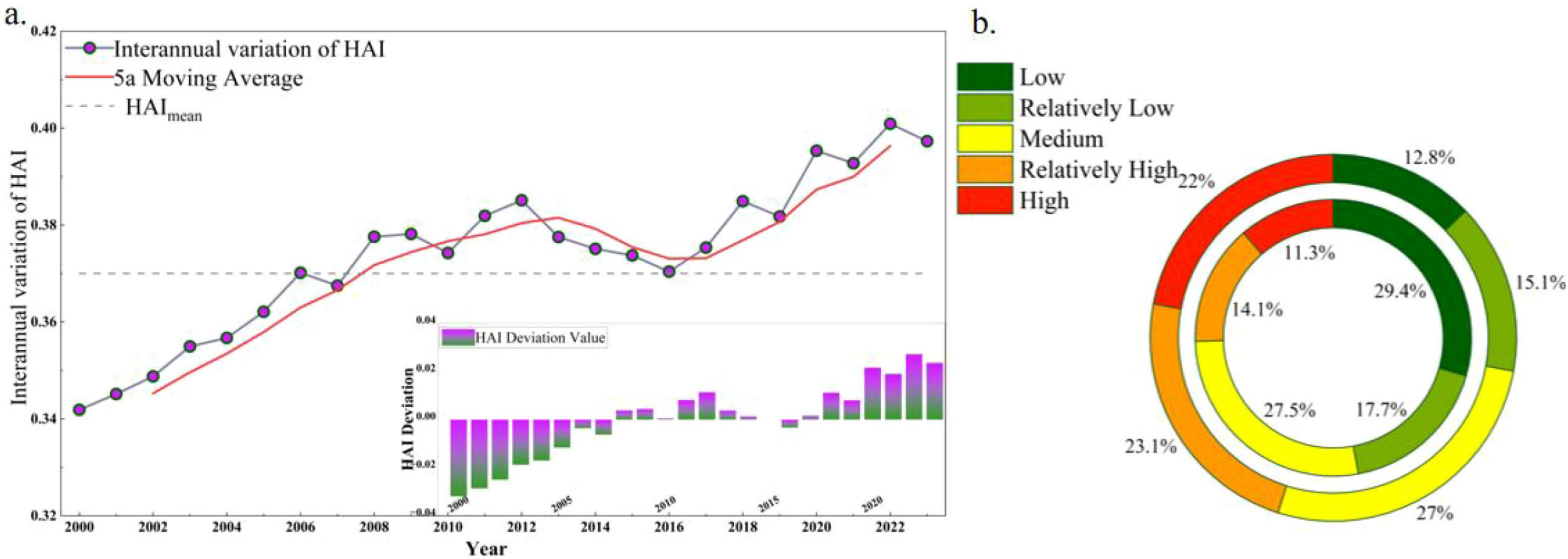
Time variation of HAI in the study area from 2000 to 2023 **(a)** is the interannual variation trend diagram of HAI index; **(b)** is the ring diagram of area proportion of HAI grades in 2000 and 2020.

HAI in the study area exhibited a consistent upward trend from 2000 to 2022 (**Figure 5**). Between 2000 and 2012, HAI experienced slight interannual fluctuations but maintained a clear upward trajectory. From 2012 to 2016, interannual variability intensified, showing alternating increases and decreases. After 2016, HAI returned to a stronger upward trajectory, and growth accelerated in the later years. The share of areas with high or very high HAI rose from 25.4% in 2000 to 45.1% in 2023, reflecting rapid expansion. These regions were marked by frequent human activities and intensive land development, underscoring the need to mitigate potential ecological pressures from high-intensity land use.

**Figure 6:**
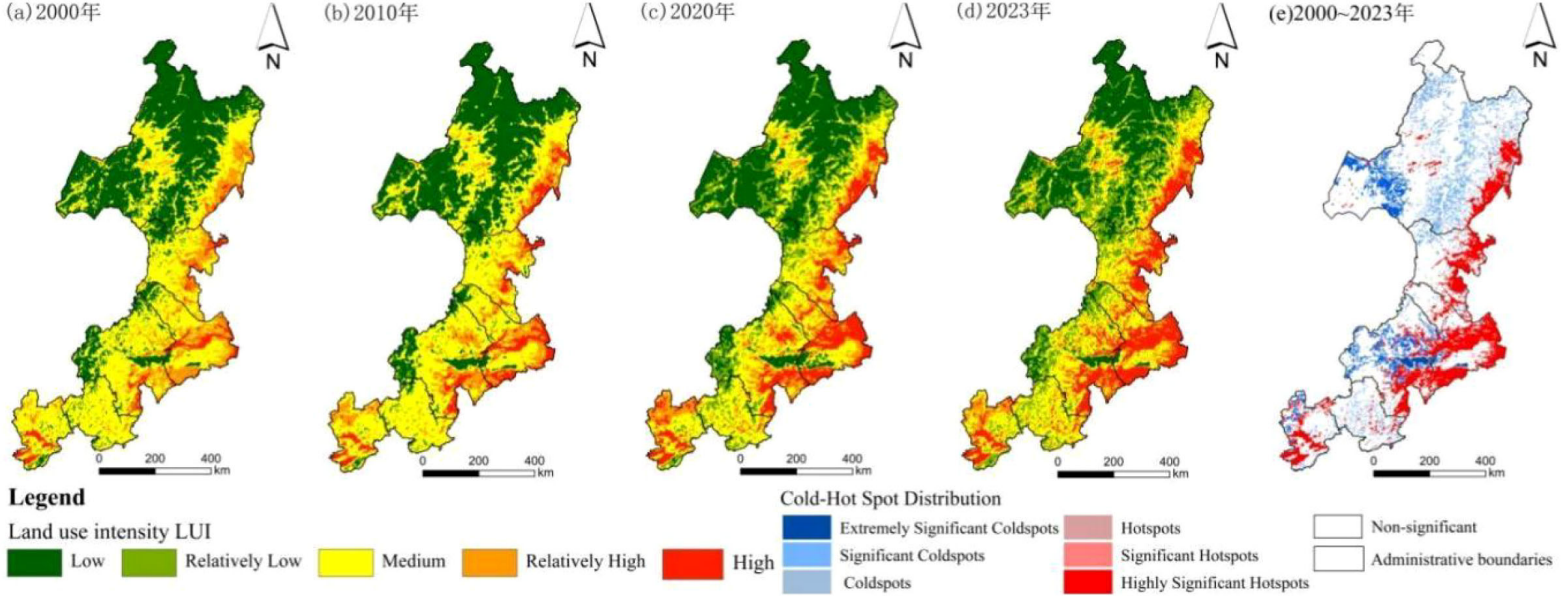
Spatial changes of HAI in the study area from 2000 to 2023.

HAI in the Daxing’anling region exhibited dynamic changes, showing a distribution pattern opposite to that of EHI (**Figure 6**). Areas with high or relatively high HAI were concentrated in the eastern plains, urban centers, and areas near major transportation corridors, forming a dispersed pattern. Moderate HAI was mainly observed in cultivated land, whereas low or very low HAI dominated forested and grassland regions in mountainous areas. In 2000, low and very low levels prevailed, with only small patches of moderate-to-high intensity use. After 2010, moderate and high levels expanded around cities and along transportation routes, indicating intensified human disturbance. By 2020, high-intensity zones expanded further, reflecting sustained growth in land development pressure. Hot spot analysis revealed that significant and highly significant increases were concentrated in the southern and eastern plains, whereas significant decreases occurred in the northern and central–western mountainous areas.

### 4.3 Analysis of the spatiotemporal relationship between EHI and HAI

#### 4.3.1 Spatial correlation analysis between EHI and HAI

**Figure 7:**
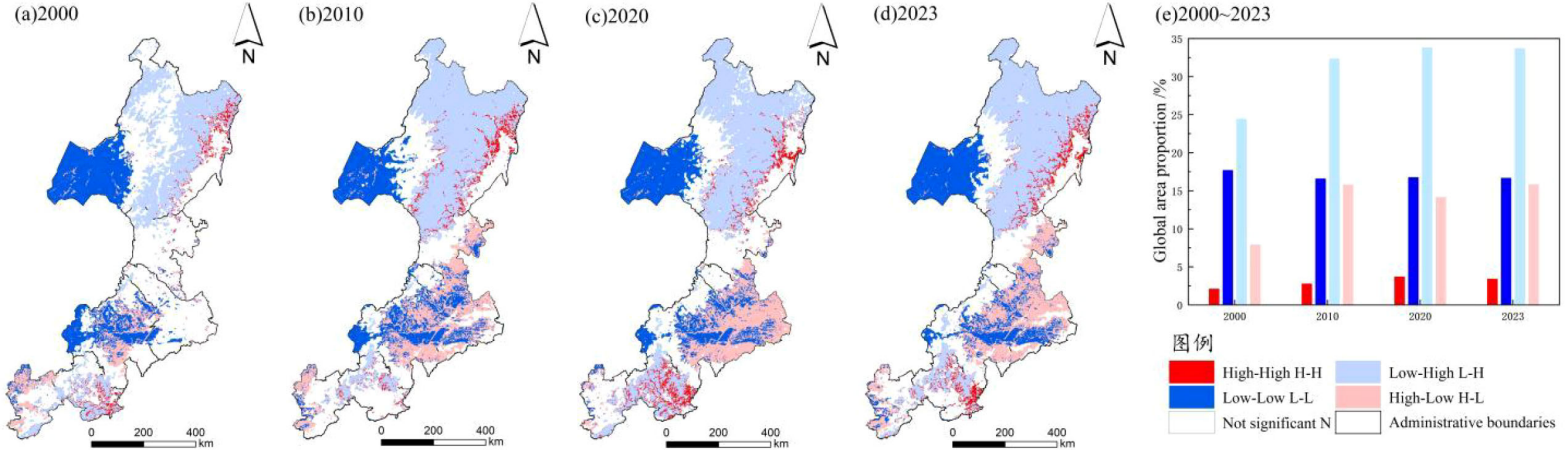
Temporal-spatial coupling of EHI and HAI (2000–2023).

At the global scale, the EHI and HAI exhibited a significant negative spatial correlation (Moran’s I< 0, p< 0.01) (**Table 5**). As shown in **Figure 7**, Moran’s I displayed a U-shaped trajectory from 2000 to 2023. The negative correlation intensified from 2010 to 2020, weakened slightly after 2020, yet the overall negative effect continued to increase. Spatial clustering of HAI and EHI in the Daxing’anling region showed clear regional differentiation. The dominant patterns were Low HAI–High EHI and High HAI–Low EHI. In the northern forests and some mountainous areas, Low HAI–High EHI clusters dominated, suggesting that high ecological health was associated with low development intensity. In contrast, the central and eastern plains were dominated by High HAI–Low EHI clusters, reflecting the negative impact of intensive development on EHI. High HAI–Low EHI clusters continued to expand across the plains, especially around farmland, towns, and major transport corridors. Meanwhile, both High HAI–High EHI and Low HAI–Low EHI clusters decreased in extent. The former appeared sporadically in the southern plains and northeastern mountains, whereas the latter contracted and fluctuated in the eastern plains and northwestern mountains. These patterns confirm that the negative coupling between EHI and land development has been further reinforced in the plains.

**Table 5:** Changes of Moran index in the study area from 2000 to 2023.

Year	Moran’s I	Z	P
2000	-0.462	-14.38	0.001
2010	-0.573	-18.72	0.001
2020	-0.646	-26.51	0.001
2023	-0.622	-17.44	0.001

#### 4.3.2 Coupled coordination analysis of EHI and HAI

Time-series analysis of the standardized coupling coordination index (**Table 6**) indicated significant improvements in Hulunbuir, Xing’an League, Tongliao, and Chifeng. Hulunbuir first increased and then declined, while Xing’an League exhibited continuous growth of 8.1%. Tongliao and Chifeng increased by 8.6% and 5.8%, respectively. Chengde remained at a consistently high level. In contrast, Zhangjiakou fluctuated, peaking at 0.761, dropping to 0.722 in 2020, and then partially rebounding to 0.745 in 2023. The regional mean showed notable interannual variability: a decline of 0.7% during 2000–2010, an increase of 5.5% during 2010–2020, and a slight adjustment to 0.845 in 2023. These results suggest that the coupling between HAI and EHI strengthened in most cities, although some fluctuations occurred.

**Table 6:** Average standardized coupling index of each city from 2000 to 2023.

Administrative region/year	2000	2010	2020	2023
Hulunbeier	0.828	0.844	0.890	0.841
Xingan	0.776	0.795	0.813	0.839
Tongliao	0.765	0.777	0.784	0.831
Chifeng	0.795	0.794	0.828	0.841
Zhangjiakou	0.667	0.761	0.722	0.745
Chengde	0.850	0.840	0.841	0.843
Overall domain	0.850	0.844	0.890	0.845

**Figure 8:**
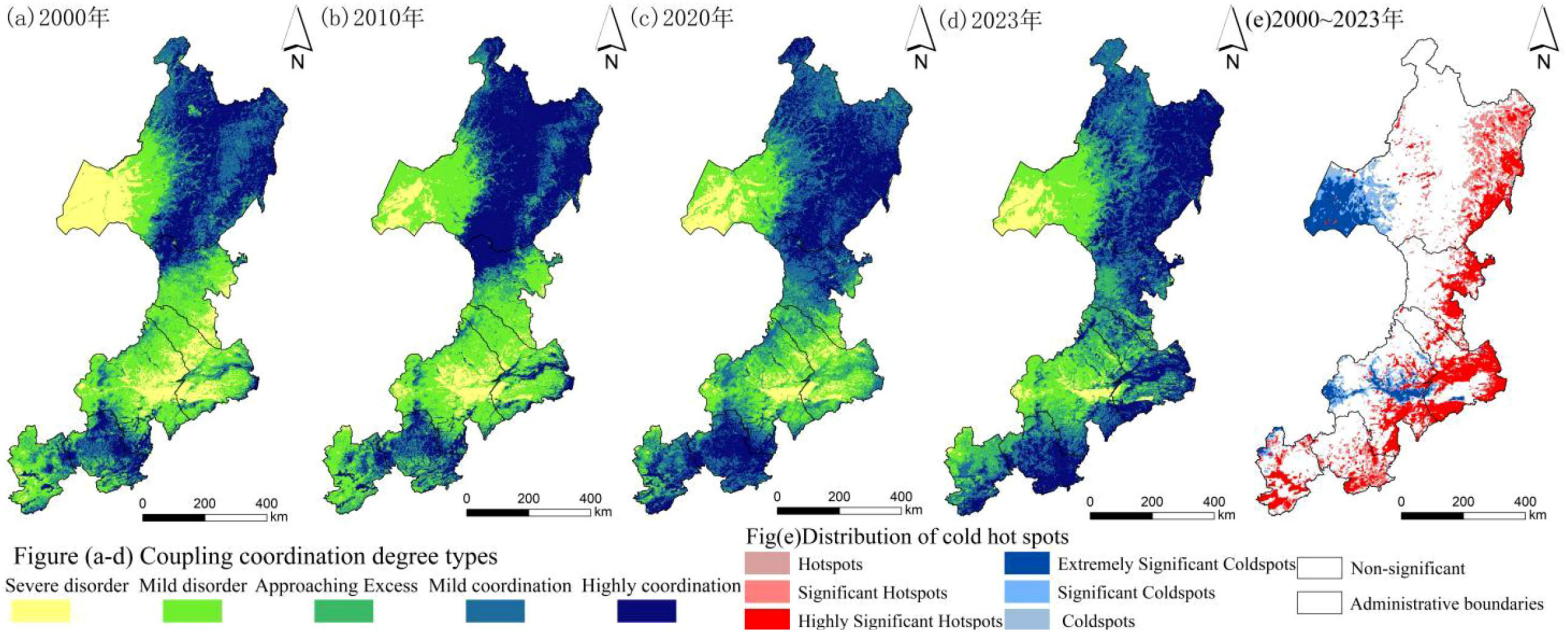
Coupling level of EHI and HAI and hot spots of coupling index change from 2000 to 2020.

The normalized coupling index was divided into five categories using ArcGIS: severe imbalance, mild imbalance, near imbalance, mild coordination, and high coordination (**Figures 8a–d**). From 2000 to 2023, the index displayed strong spatial variability, forming clusters of both high and low values. Low-value areas were mainly distributed in western Hulunbuir, southeastern Xing’an League, most of Tongliao and Chifeng, and the western and northern parts of Zhangjiakou. These clusters reflected frequent human activity and ecological degradation beyond carrying capacity. In contrast, coordinated areas were mainly located in northern and eastern Hulunbuir, northeastern Xing’an League, southeastern Tongliao, central and southern Chengde, and southern Zhangjiakou. These regions were characterized by low human disturbance, high forest cover, and well-preserved ecosystem services. Notably, high-value clusters of coordination expanded over the study period. Significance analysis of changes in the coupling index (**Figure 8e**) revealed patchy cold spots (≥95% confidence) where the index declined, mainly in western Hulunbuir and central and western Chifeng, with scattered areas in Tongliao and Zhangjiakou. These locations corresponded to high human disturbance and rapid urban expansion. In contrast, significant increases (≥95% confidence) were concentrated in the eastern plains, including southeastern Hulunbuir, eastern Xing’an League, central and southeastern Tongliao, central and southern Chifeng, southern Chengde, and southern Zhangjiakou.

#### 4.3.3 Functional correlation between EHI and HAI

From a temporal perspective, the four quadrants of the Daxing’anling region underwent substantial changes between 2000 and 2023. The proportions of Quadrants I and III decreased by 3.7% and 7.1%, respectively, while Quadrants II and IV increased by 6.9% and 3.6%. Quadrants II and IV covered larger areas and emerged as the dominant types in the four-quadrant framework. To further evaluate the coupling between EHI and HAI, the mean normalized coupling index was calculated for each quadrant from 2000 to 2023 (**Table 7**). The 20-year mean values were ranked as follows: Quadrant I (0.836) > Quadrant IV (0.745) > Quadrant II (0.688) > Quadrant III (0.653). Temporal variation was also evident. In Quadrant I, the coupling coordination index fluctuated, increasing from 0.725 in 2000 to a peak of 0.893 in 2020, and then declining slightly to 0.884 in 2023. In Quadrant II, the index increased gradually from 0.601 in 2000 to 0.764 in 2010, and then stabilized between 0.68 and 0.70. In Quadrant III, the index displayed a U-shaped trajectory, decreasing from 0.685 in 2000 to a minimum of 0.597 in 2010, and then gradually recovering to 0.716 in 2023. In Quadrant IV, the index remained relatively stable between 0.70 and 0.75, with minimal fluctuations.

**Table 7:** The average coupling index of each quadrant from 2000 to 2023.

Quadrant/year	2000	2010	2020	2023
Quadrant I	0.725	0.844	0.893	0.884
Quadrant II	0.601	0.764	0.689	0.701
Quadrant III	0.685	0.597	0.622	0.716
Quadrant IV	0.758	0.741	0.738	0.747

**Figure 9:**
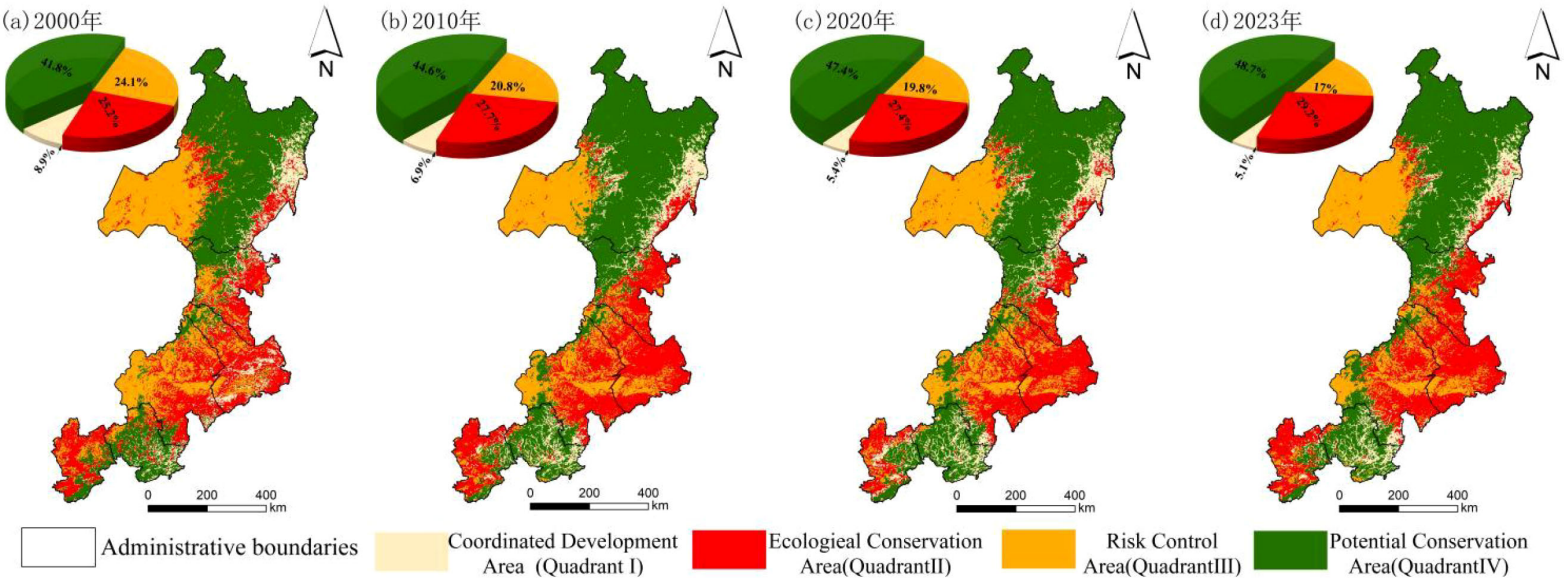
Four-quadrant model of EHI and HAI in the study area.

As shown in **Figure 9**, spatial differentiation among the four ecological zones of the Daxing’anling region increased markedly from 2000 to 2023 (**Figure 9**). Quadrant I, the Coordinated Development Zone, was concentrated along the eastern margins of Hulunbuir City and Hinggan League. This zone exhibited strong coupling between HAI and EHI. Land use efficiency in the plains was high, with strong capacity for risk resistance. However, its share of the total area declined steadily, from 8.9% in 2000 to 5.1% in 2023. Quadrant II, the Ecological Management Zone, was mainly distributed in eastern Hinggan League, central and eastern Tongliao and Chifeng, and southwestern Zhangjiakou. Because of extensive construction land and frequent human activities, its share increased from 25.2% in 2000 to 29.2% in 2023. Quadrant III, the Risk Prevention Zone, was located in the western and central-western margins of Hulunbuir, where cropland was interspersed with forests and grasslands. In this zone, the coupling between HAI and EHI was weak, and its share declined steadily from 24.1% in 2000 to 17.0% in 2023. Quadrant IV, the Potential Conservation Zone, extended across the northern and southeastern parts of the Daxing’anling region, including northern and central Hulunbuir, northwestern Hinggan League, and most of Chengde. Dominated by forests and grasslands with minimal human disturbance, this zone expanded from 41.8% in 2000 to 48.7% in 2023.

### 4.4 Driving factor analysis

#### 4.4.1 Analysis of regression model based on XGBoost

The XGBoost regression model developed in this study showed robust predictive performance in both the training and testing datasets (**Table 8**). For EHI predictions, the coefficient of determination (R^2^) was higher in the training set (0.761) than in the testing set (0.728). The root mean square error (RMSE) of the testing set was 0.122 higher than that of the training set, whereas the mean absolute error (MAE) differed by only 0.024. These results indicate stable predictive performance. For HAI, the training set produced a higher R^2^ (0.896) than the testing set (0.763). The RMSE was 0.159 in the testing set and 0.111 in the training set, while the MAE remained below 0.06. For coupling coordination, the R^2^ reached 0.887 in the training set and 0.774 in the testing set. The RMSE of the testing set was 0.02 lower than that of the training set, whereas the MAE differed by 0.049. Statistical tests confirmed that residuals across all indicators met the assumptions of normality and homoscedasticity. These findings support the reliability of the model in adapting to the data structure.

**Table 8:** Test set and validation set results based on XGBoost model.

Model name	Training set			Test set		
	R^2^	RMSE	MAE	R^2^	RMSE	MAE
Ecosystem Health Index	0.761	0.162	0.120	0.728	0.284	0.144
Human Activity Intensity	0.896	0.111	0.036	0.763	0.159	0.102
Coupling Coordination Degree	0.887	0.139	0.047	0.774	0.153	0.096

#### 4.4.2 Explanatory analysis with SHAP model

**Figure 10:**
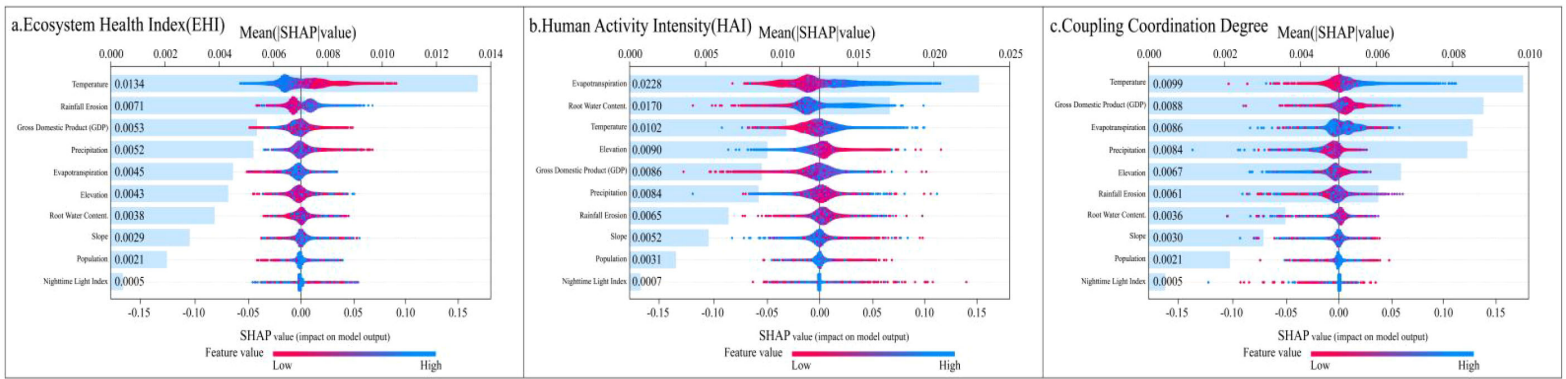
Analysis of SHAP features of the dependent variable ecological model.

The XGBoost machine learning algorithm was used to build predictive models, while the SHAP interpretability framework was incorporated to systematically examine the drivers of the EHI, HAI, and their Coupling Coordination Degree (**Figure 10**). Within this framework, changes in the three indices were defined as dependent variables (Y), whereas ten natural and socio-economic factors were considered independent variables (X). This approach allowed evaluation of the relative contributions of the factors and the mechanisms through which they influenced the outcomes. Bee-swarm plots were applied to visualize the results. Features with SHAP values below zero were interpreted as negative contributors, whereas values above zero were considered positive contributors. The SHAP-derived importance ranking identified distinct key factors for each index. For EHI, PET, root-zone soil moisture, and temperature were the most influential factors, contributing 27.29%, 14.46%, and 10.79%, respectively. For HAI, temperature, gross domestic product (GDP), and PET were the leading contributors, accounting for 24.91%, 18.57%, and 11.14%, respectively. For coupling coordination, temperature, rainfall-induced erosion, and GDP were the main determinants, contributing 17.15%, 15.25%, and 14.90%, respectively.

**Figure 11:**
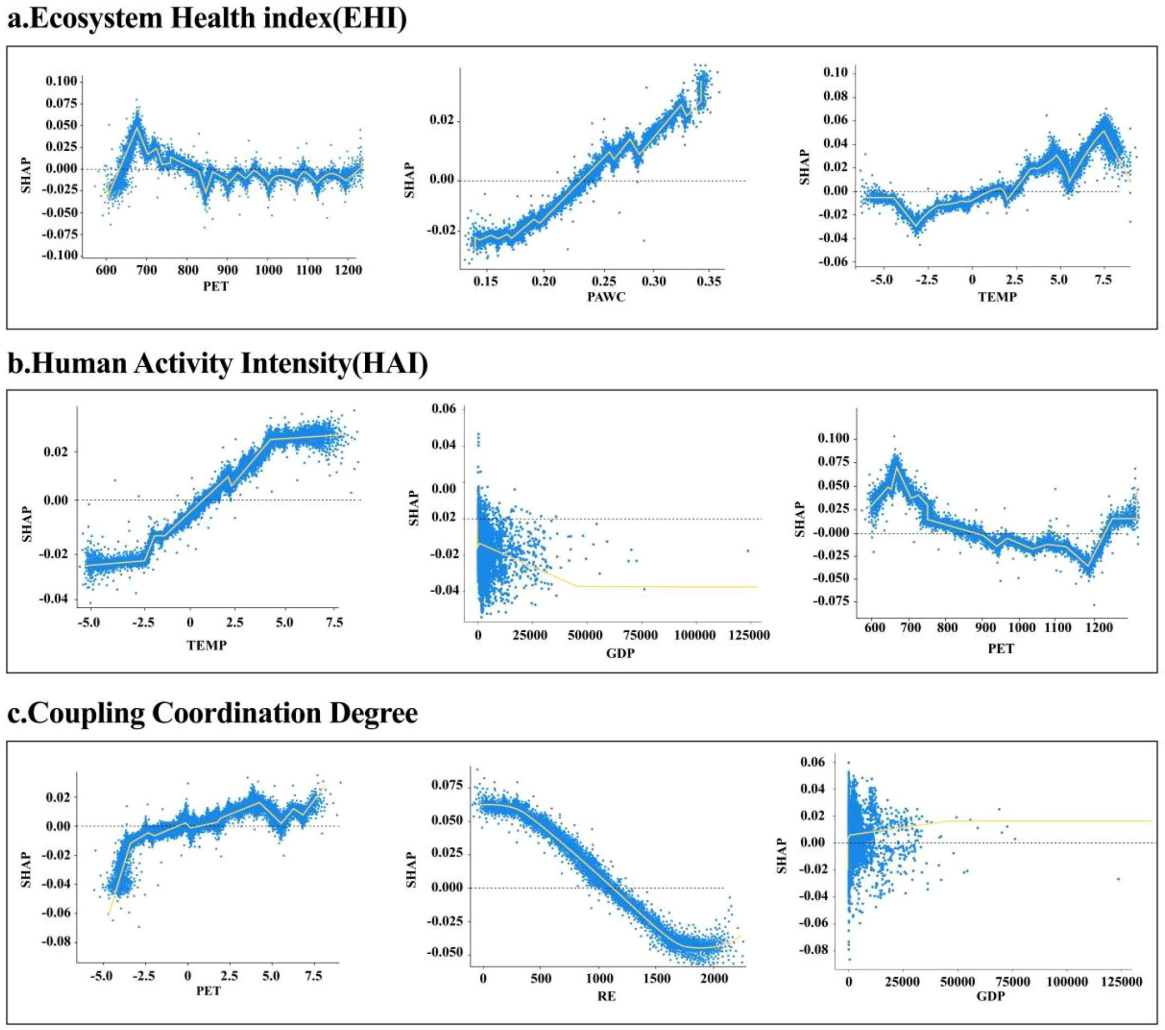
Analysis of influencing factors of ecological model based on SHAP dependency graph. **(a)** Ecosystem Health Index (EHI), **(b)** Human Activity Intensity (HAI), and **(c)** Coupling Coordination Degree, in accordance with journal guidelines.

The SHAP dependence plots revealed the marginal effects of each variable on ecological vulnerability and clarified the complex relationships between vulnerability and its key drivers (**Figure 11**). Overall, the dependent variables showed largely linear responses to the main predictors, but threshold effects were apparent, with critical values clustering near zero. PET was identified as a major determinant of EHI. At low levels, enhanced plant water retention produced positive SHAP values, whereas values declined and became negative when PET exceeded 800 mm, indicating potential adverse effects. Plant-available water showed an opposite trend: SHAP values were negative at low levels but stabilized and turned positive with increasing water availability, suggesting that higher soil moisture supports EHI. Temperature also played a critical role. Below 0°C, SHAP values fluctuated markedly, but above this threshold, contributions to EHI became increasingly positive. For HAI, temperature was again the most influential driver. SHAP values were positive above 0°C, indicating that warmer conditions promoted higher activity levels. Gross domestic product (GDP) also contributed positively: SHAP values fluctuated at low GDP levels but rose and stabilized as GDP increased, reflecting the facilitative role of economic growth. In contrast, PET showed a negative relationship, with SHAP values turning negative around 850 mm, suggesting that excessive PET suppressed HAI. For the coupling coordination degree, temperature showed a clear positive association. Values decreased when temperature was below 0 °C but rose steadily within the 0–7.5 °C range. Rainfall-induced erosion had the opposite effect, as SHAP values became negative when erosion exceeded 1000 MJ·mm·(hm^2^·h·a)^−1^, indicating a persistent reduction in coordination with increasing erosion. GDP displayed a non-linear effect: low values were positively correlated with coordination, but once GDP exceeded 10 million yuan/km^2^, the relationship turned negative and stabilized, with SHAP values showing a gradual decline.

## 5 Discussion

### 5.1 Spatiotemporal variation of EHI and HAI

The spatiotemporal dynamics of the EHI and HAI in the forest–grassland ecotone of Northeast China from 2000 to 2023 were strongly associated with the natural conditions of the cold-temperate transition zone and regional development patterns. Overall, EHI increased by 25.1%, consistent with Xu et al., who reported a 19.8% rise in ecosystem services in the Daxing’anling region. This improvement was likely driven by the Natural Forest Protection Program and the Grain-for-Green Project launched after 2000 (Shim and Choi, 2024). These policy-driven land-use changes enhanced vegetation cover and ecosystem stability, thereby improving ecosystem vitality and service provision, which are core components of EHI. A distinct north–high, south–low gradient of EHI was observed, supporting previous findings on ecosystem distribution in the region. This spatial pattern reflects the underlying climatic and ecological gradients of the cold-temperate transition zone, where lower temperatures, higher forest coverage, and reduced human disturbance in the northern mountainous areas favor higher primary productivity and ecosystem resilience. In contrast, HAI followed a plain–high, mountain–low distribution. Development intensity was greater in the southern plains than in the northern forests, mainly due to cropland expansion and urban land growth, a pattern consistent with findings from Fu et al. in the Shaanxi section of the Yellow River Basin. This spatial pattern is consistent with previous studies that employed LUCC-based indicators to characterize HAI or land-use disturbance in northern China. Compared with composite indices such as the Human Footprint Index, which emphasize static infrastructure and population proxies, the HAI framework used in this study places greater emphasis on land-use disturbance intensity and its spatial heterogeneity. Similar land-use weighting strategies have been adopted in studies focusing on forest–grassland ecotones and ecologically fragile regions (Yan L. et al., 2025; Peng et al., 2017), supporting the applicability of the adopted approach in the forest–grassland ecotone. Spatial autocorrelation analysis showed (Zhao et al., 2025) that the global Moran’s I for EHI and HAI declined from –0.462 in 2000 to –0.622 in 2023 (p< 0.01), indicating that their negative coupling became significantly stronger. High EHI–low HAI clusters remained concentrated in the northern primary forests, where intact forest cover was preserved through strict protection (Fu et al., 2025; Xu et al., 2025). Conversely, low EHI–high HAI clusters dominated the flat agro-pastoral ecotones, where intensive land-use practices disrupt vegetation structure and soil stability, increasing ecosystem vulnerability and reinforcing the spatial trade-off between human activities and ecosystem health in cold-temperate environments.

### 5.2 Spatial differentiation of reciprocal feed mechanism and regional regulation policy suggestions

Between 2000 and 2023, the coupling coordination degree in the forest–grassland ecotone of Northeast China showed a distinct spatial gradient. This finding is consistent with the broader recognition of spatial heterogeneity in human–environment interactions in ecotones reported by Li et al. (2023) (Zhao et al., 2024). It also offers a more refined description of subregional patterns and temporal changes. In the northern forested areas, such as Hulunbuir and Hinggan League, coordination remained high (D > 0.8). By contrast, in the southern plains, including Tongliao and Chifeng, coordination persisted at a critical level (D = 0.6–0.8). This reflected growing conflicts between human activity and ecological carrying capacity. This north–high, south–low pattern can be attributed to differences in ecosystem structure, land-use intensity, and adaptive capacity. Northern regions are dominated by continuous forest cover with lower disturbance intensity, which buffers climatic variability and maintains stable ecosystem functioning. In contrast, southern plains are characterized by intensive agriculture and urban expansion, which amplify ecological sensitivity to both climatic stress and anthropogenic disturbance, leading to reduced coordination between human activities and ecosystem health. The north–high, south–low pattern supports the Pressure–State–Response framework proposed by Zhang et al. (2019) for fragile ecosystems in northern China (Pang et al., 2025). These patterns correspond closely with the spatial diagnosis provided by the four-quadrant model. The Coordinated Development Zone (Quadrant I) declined in area but retained strong synergy between land-use efficiency and ecosystem health. This indicates that even in highly coordinated systems, increasing development intensity may gradually erode ecological resilience if management responses lag behind pressure accumulation. This region requires attention to potential hidden disturbances, such as tourism development. Future strategies should involve ecological compensation mechanisms and restrictions on uncontrolled land expansion. The Ecological Management Zone (Quadrant II) expanded slightly. Intensive farming in this zone has accelerated soil erosion. Strict enforcement of the Grain-for-Green policy is required, along with the promotion of crop–pasture rotation and ecological engineering to enhance vegetation cover. The Risk Prevention Zone (Quadrant III) was defined by ecological fragility and dual-low conditions (EHI< 0.4, HAI< 0.3). Priority should be placed on establishing ecological redlines, controlling grazing intensity, and introducing cold-resistant grass species to restore degraded pastures. The Potential Conservation Zone (Quadrant IV) expanded and benefited from favorable ecological conditions. In this zone, the Natural Forest Protection Program should be strengthened, mineral exploitation prohibited, and mechanisms for realizing ecological product values explored (Liu J. et al., 2025; Liu X. et al., 2025). Accordingly, targeted regulatory strategies are proposed. In mountainous regions, a forest–grass–water integrated optimization plan should be implemented. Adjustments in tree species composition should be made to enhance water conservation. In the plains, a compact city plus ecological corridor approach should be adopted. Expansion of construction land should be confined to intensive development areas. Permanent farmland protection boundaries should be delineated to restrict cropland expansion and safeguard ecological space (Abdullah and Mustafa, 2025). By linking coupling coordination patterns to underlying differences in ecosystem resilience and land-use pressure, these differentiated strategies provide a process-informed basis for coordinated governance in cold-temperate ecotones.

### 5.3 Analysis of model driving factors and discussion on model applicability

In recent years, studies on the driving mechanisms of human–environment systems in ecologically fragile regions have mainly relied on traditional approaches such as geographical detectors and geographically weighted regression. Although effective in identifying factor contributions, these methods cannot capture nonlinear interactions or critical thresholds (Cai et al., 2025; Zhang et al., 2025). To address this limitation, interpretable machine-learning approaches, particularly the XGBoost–SHAP framework, have been increasingly applied in ecological research. Previous studies primarily emphasized factor importance ranking or interaction identification. For example, Huang et al. (2025) applied XGBoost–SHAP to classify ecological functional zones in Chengdu, while Wu et al. (2025) highlighted nonlinear governance effects on ecological resilience (Yu and Li, 2024; Huang et al., 2025). Building on these studies, the present work extends the framework by focusing on response thresholds and direction shifts, and by jointly analyzing EHI, HAI, and their coupling coordination degree. Accordingly, SHAP-derived nonlinear breakpoints are interpreted as indicators of ecological constraint transitions rather than purely statistical artifacts, supported by studies showing that climate-driven changes in soil nutrient availability, plant functional traits, and biomass allocation mediate ecosystem responses (Gao et al., 2023; Gong et al., 2023). The advantages of this approach are threefold. First, it enables explicit identification of climatically meaningful thresholds. SHAP analysis indicates that PET exceeding 850 mm significantly inhibits EHI, while GDP above 10 million yuan km^−2^ shifts the marginal effect on coupling coordination from positive to negative. Ecologically, the PET threshold reflects a transition from energy-limited to water-limited conditions, under which increased atmospheric evaporative demand intensifies plant water stress, constrains stomatal regulation, and reduces carbon assimilation, consistent with previous studies linking high evaporative demand to productivity and ecosystem service reductions (Taelman et al., 2016). These processes weaken ecosystem vitality, represented by NPP-based indicators in the EHI framework, and propagate to ecosystem service proxies such as carbon storage and soil retention. Second, the framework clarifies nonlinear interactions among key climatic drivers. Temperature emerges as a central factor: low temperatures (< 0 °C) suppress vegetation growth by shortening the growing season and limiting physiological activity, whereas higher temperatures (> 0 °C) enhance net primary productivity but simultaneously promote urbanization and agricultural expansion. These contrasting responses reflect how climatic forcing shapes ecosystem health through soil moisture availability, plant functional traits, and biomass allocation, generating nonlinear NPP and service outcomes (Pan et al., 2021). Third, differentiated driving dimensions are observed across system components. Natural factors primarily regulate EHI variation, socio-economic variables primarily shape HAI, and coupling coordination is jointly governed by the nonlinear interaction between rainfall erosivity and GDP. Importantly, HAI should not be viewed solely as an external pressure. Land-use change, forest management, and agricultural intensification can modify vegetation structure, canopy properties, and soil water conditions, thereby altering ecosystem sensitivity to climatic drivers. In systems where ecosystem vitality is represented by remotely sensed NPP, these management-induced structural differences can amplify or dampen vegetation responses to temperature and PET. Moreover, different land-cover or management contexts (e.g., planted vs. natural forests) exhibit distinct NPP responses to climate, highlighting the need to consider land-use type when interpreting climatic effects (Pielke et al., 2011; Pongratz et al., 2021). This limitation is acknowledged and suggests directions for future research. Accordingly, the nonlinear coupling behavior identified by SHAP reflects both pressure effects and human-mediated regulation of ecosystem sensitivity and resilience in forest–grassland ecotones. Overall, by explicitly linking SHAP-identified nonlinear thresholds to EHI components and underlying ecosystem structure–function relationships, this study strengthens the ecological interpretability of machine-learning attribution results and provides a robust basis for ecological restoration and sustainable development in cold-temperate forest–grassland ecotones.

### 5.4 Research deficiencies and future improvement directions

This study employed an ensemble modeling approach to examine the spatiotemporal coupling and threshold effects between EHI and HAI in the forest–grassland ecotone of Northeast China, providing a quantitative framework for regional ecological governance. However, several limitations should be acknowledged (Hao et al., 2022). First, the spatial heterogeneity of policy effects was not explicitly incorporated into the modeling framework. Although major ecological restoration programs were discussed qualitatively, their localized implementation intensity and temporal variability could not be fully captured, which may introduce uncertainty in attributing EHI improvements to specific driving mechanisms. Second, the absence of quantitative information on micro-ecological processes, such as soil carbon–nitrogen cycling, nutrient availability, and microbial activity, constrains the mechanistic interpretation of climate–ecosystem linkages. As a result, the identified nonlinear thresholds primarily reflect system-level responses rather than explicit process-based transitions at the soil–plant interface. Third, the use of grid-based datasets with relatively coarse spatial resolution may underestimate fine-scale human disturbances and management practices, thereby limiting the ability to fully resolve local feedbacks between land use, productivity, and ecosystem health. Taken together, these limitations suggest that future research should move toward a more process-informed integration of remote sensing, ecological observations, and policy data. Incorporating soil biogeochemical measurements, vegetation functional trait information, and stratified analyses across land-use and management types would help bridge the gap between statistical attribution and ecological mechanism. Future studies should also refine the coupling coordination framework, optimize indicator selection, and improve data precision (Wang Z. et al., 2022). These efforts will strengthen the ecological interpretability of threshold responses and provide more robust scientific evidence to support conservation planning and sustainable development in cold-temperate ecotones.

## 6 Conclusion

Using multi-source remote sensing data from 2000 to 2023, this study developed an Assessment–Coupling–Driving framework to examine the spatiotemporal interactions between EHI and HAI in the forest–grassland ecotone of Northeast China. Over the study period, EHI increased from 0.388 to 0.485, with low-health areas declining from 49.8% to 32.4%, while HAI rose steadily, with the regional mean increasing by 16.3% and high-intensity zones expanding from 25.4% to 45.1%. Spatial patterns revealed a north–high, south–low gradient for EHI and higher human activity in plains than in mountains, with pronounced spatial trade-offs indicated by negative Global Moran’s I values. Hotspot analysis identified low HAI–high EHI synergy zones in the north and high HAI–low EHI conflict zones in the southern plains, highlighting spatial heterogeneity in human–environment interactions. The coupling coordination degree remained generally coordinated, though spatial differentiation intensified, with the Coordinated Development Zone decreasing by 3.7% and the Ecological Management Zone increasing by 6.9%. Using the XGBoost–SHAP model, we identified nonlinear effects and critical thresholds of key climatic and socio-economic drivers. PET and low temperatures negatively influenced EHI, while plant-available water promoted ecological stability. Thresholds such as PET > 850 mm or GDP > 10–12 million yuan/km^2^ delineated points where ecosystem or coupling responses shifted sharply, reflecting climate–human–ecosystem interactions. Overall, temperature and rainfall erosivity emerged as dominant drivers, and human activities were shown to modulate ecosystem sensitivity to climatic factors, highlighting that ecosystem responses are shaped by complex nonlinear interactions and threshold effects. These findings advance mechanistic understanding of human–environment coupling in ecologically fragile regions and provide a quantitative basis for differentiated regional management and ecological restoration strategies.

## Data Availability

The original contributions presented in the study are included in the article. Further inquiries can be directed to the corresponding author.
